# Correlations of systemic immune-inflammation index and systemic inflammation response index with the risk for early-onset post-stroke depression in patients with minor stroke: a prospective observational study

**DOI:** 10.3389/fneur.2026.1760381

**Published:** 2026-07-06

**Authors:** Wei Zhao, Mingzhu Deng, Zhen Wang, Guohua He, Wei Xu, Tieqiao Feng, Jian Peng, Kangping Song, Ling Xiao, Fangyi Li

**Affiliations:** 1 Department of Neurology, The Affiliated Changsha Central Hospital, Hengyang Medical School, University of South China, Changsha, Hunan, China; 2 Department of Neurology, The Second People’s Hospital of Hunan Province (Brain Hospital of Hunan Province), Changsha, Hunan, China

**Keywords:** depression, inflammation, post-stroke depression, systemic immune-inflammation index, systemic inflammation response index

## Abstract

**Background:**

Inflammation plays a pivotal role in the pathophysiology of post-stroke depression (PSD). However, the relationship between novel systemic inflammatory indices-the systemic immune-inflammation index (SII) and systemic inflammation response index (SIRI)-and early-onset PSD remains inadequately explored.

**Methods:**

Early-onset PSD was diagnosed 2 weeks after acute ischemic stroke (AIS). Depression severity was assessed using the 17-item Hamilton Depression Rating Scale (HAMD-17); patients with scores ≥7 were classified into the early-onset PSD group. Spearman rank correlation analysis was performed to evaluate associations of SII and SIRI with HAMD-17 scores across all participants. Binary logistic regression was used to examine the independent associations of SII and SIRI with early-onset PSD. Receiver operating characteristic (ROC) analysis was employed to assess the SII and SIRI capacity to differentiate early-onset PSD.

**Results:**

Of the 1,113 prospectively enrolled patients, 372 (33.42%) were diagnosed with early-onset PSD. HAMD-17 scores showed significant positive correlations with SII (*r* = 0.440, *p* < 0.001) and SIRI (*r* = 0.418, *p* < 0.001). Both SII (OR = 1.762, 95% CI: 1.261–1.946, *p* < 0.001) and SIRI (OR = 1.672, 95% CI: 1.348–1.932, *p* = 0.004) emerged as independent predictors of early-onset PSD. The areas under the curve (AUC) for SII, SIRI, and their combination were 0.767, 0.718, and 0.807, respectively.

**Conclusion:**

SII and SIRI may serve as independent risk factors for early-onset PSD. These indices offer potential utility for risk stratification and could inform prevention strategies and prognosis management in this patient population.

## Introduction

Post-stroke depression (PSD), clinically defined as a depressive state following stroke onset, represents a prevalent neuropsychiatric complication of ischemic stroke. Its core manifestations encompass anhedonia, depressed mood, diminished interest, and feelings of worthlessness (1). In recent years, the growing population of stroke survivors has led to a substantial increase in PSD cases, with approximately one-third of individuals experiencing depression after stroke (2). Early-onset PSD is specifically characterized by the emergence of depressive symptoms within the first 2 weeks after an acute stroke (1, 3, 4). Compared to late-onset PSD, this subtype demonstrates a higher incidence of depressive features and is associated with a significantly elevated risk of adverse clinical outcomes (5). Currently, the diagnosis of PSD remains challenging in clinical practice. It is estimated that only about 5% of stroke patients receive a formal PSD diagnosis and subsequent treatment (6, 7). This underscores the critical need for proactive symptom screening in post-stroke populations to reduce the rate of underdiagnosis.

Inflammation plays a pivotal role in the pathophysiology of PSD (8). Stroke, particularly ischemic stroke, initiates a cascade of inflammatory responses (9), leading to the activation of microglia and astrocytes. These activated cells release cytokines, chemokines, matrix metalloproteinases, reactive oxygen species, and nitric oxide, which collectively exacerbate brain tissue injury and compromise blood–brain barrier (BBB) integrity (10). In recent years, novel composite hematological indices-the systemic immune-inflammation index (SII) and systemic inflammation response index (SIRI)-have been recognized as sensitive markers of systemic inflammation. A growing body of evidence supports the relevance of SII in cerebrovascular diseases. A meta-analysis demonstrated a significant association between elevated SII levels and poor stroke outcomes (11). SII has also been identified as an independent risk factor for stroke-associated pneumonia in patients with intracerebral hemorrhage and is correlated with unfavorable prognoses (12). A cross-sectional study linked SII to cerebral small vessel disease, underscoring its prognostic value (13). Previous research has confirmed that increased serum SII levels are closely related to the severity of carotid artery stenosis (14). Additionally, two recent large-scale cross-sectional studies reported associations between elevated SII and stroke risk in U. S. populations (15, 16). Several studies have explored the relationship between SII and depression. One investigation found a positive correlation between abnormal baseline SII and anxiety/depression scores in COVID-19 survivors (17). Another study linked SII to suicide attempts among adolescents with major depressive disorder during the COVID-19 pandemic (18). Higher SII levels have also been associated with worse prognosis in patients with major depressive disorder (19). A cross-sectional study in a U. S. cohort further demonstrated a significant association between SII and post-stroke depression (20). To date, only one prospective study has supported a significant correlation between elevated admission SII and PSD (21). However, its relatively small sample size necessitates validation in larger cohorts. Consequently, the role of SII in early-onset PSD warrants further investigation. SIRI has recently emerged as a novel marker of systemic inflammatory status and has been significantly associated with stroke. Previous studies have shown that SIRI is independently associated with a history of stroke (22, 23). A meta-analysis indicated that acute ischemic stroke patients with higher SIRI levels upon admission had poorer functional outcomes at 3 months (24). Elevated SIRI is also linked to increased mortality, higher risk of sepsis, and greater stroke severity (25), and serves as a risk factor for ischemic stroke recurrence (26). Nevertheless, direct investigations into the association between SIRI and early-onset PSD remain scarce.

Early-onset PSD is linked to worse prognosis (27), but its association with inflammatory indices SII and SIRI is poorly defined. This prospective study investigates the relationship between SII, SIRI, and early-onset PSD.

## Materials and methods

### Study approval and participant enrollment

**Figure 1:**
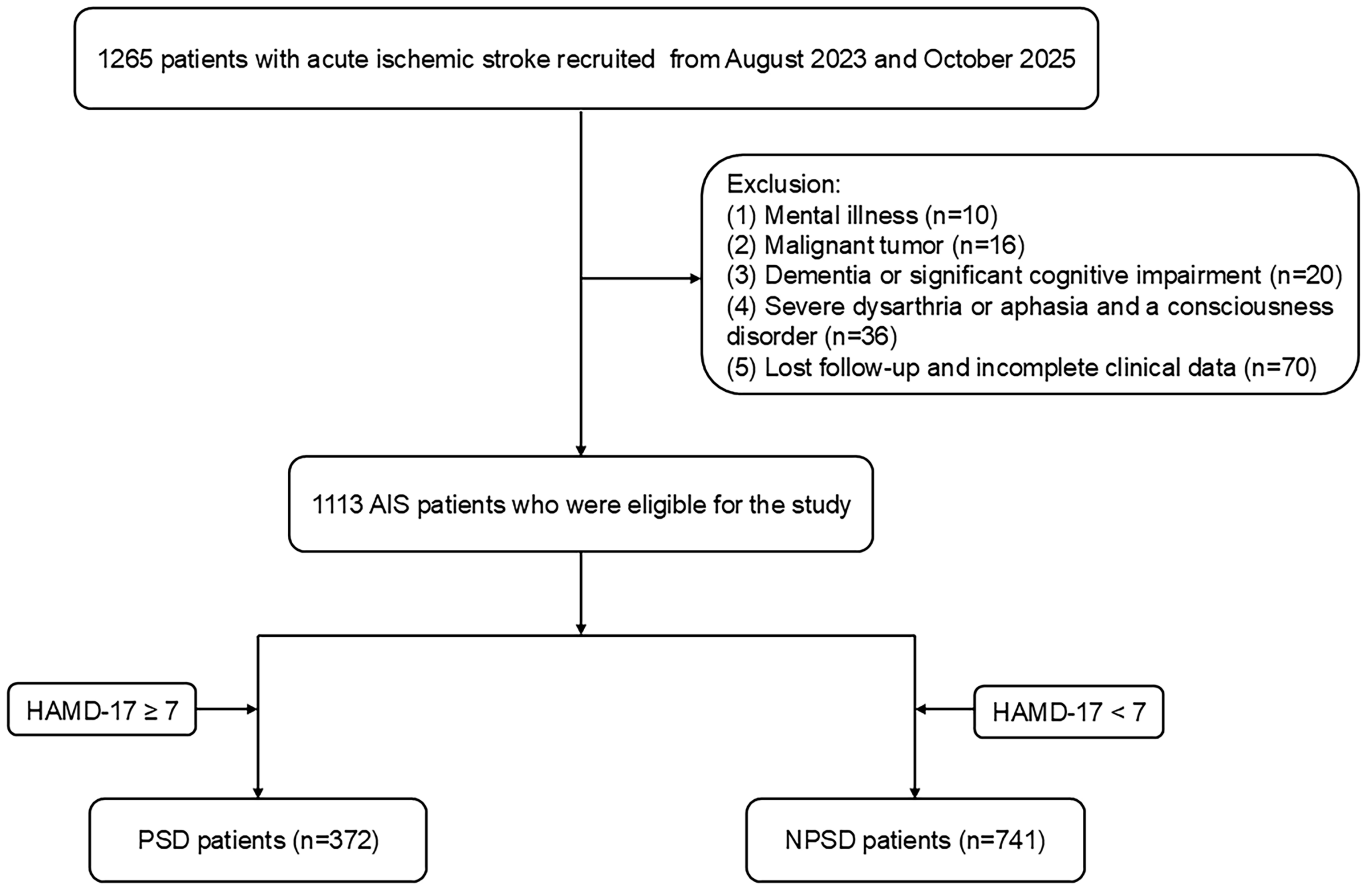
The flow diagram for the investigation. AIS, acute ischemic stroke; PSD, post-stroke depression, NPSD, Non-post-stroke depression.

This study received ethical approval from the Institutional review board of Changsha Central Hospital. From August 2023 to October 2025, we prospectively enrolled consecutive patients diagnosed with acute ischemic stroke (AIS) at Changsha Central Hospital. Inclusion criteria were as follows: (1) age between 18 and 85 years; (2) diagnosis of ischemic stroke according to the 2018 Chinese guidelines for the diagnosis and treatment of acute ischemic stroke (28); and (3) hospital admission within 72 h of symptom onset. Exclusion criteria included: (1) severe dysarthria, aphasia, or impaired consciousness precluding reliable assessment; (2) pre-stroke diagnosis of dementia or significant cognitive impairment; (3) severe cardiac, hepatic, or renal insufficiency; (4) pre-existing psychiatric disorders (e.g., depression) or use of psychotropic medications; (5) history of other central nervous system diseases (e.g., Parkinson’s disease, epilepsy); (6) active malignancy; and (7) loss to follow-up or incomplete clinical data. During the study period, a total of 1,113 AIS patients were enrolled (Figure 1).

### Data collection

Upon admission, all participants underwent standardized assessment of demographic characteristics (age, sex), anthropometric measures (body mass index, BMI), vascular risk factors (diabetes mellitus, hypertension, atrial fibrillation, coronary artery disease, current smoking, and alcohol consumption), and routine laboratory tests. Current smoking was defined as consistent consumption of ≥10 cigarettes per day for at least 5 years prior to enrollment. Similarly, current alcohol consumption was defined as regular intake of ≥20 grams of ethanol daily for a minimum of 5 years.

Stroke severity was evaluated by experienced neurologists using the National Institutes of Health Stroke Scale (NIHSS), with scores recorded within 24 h of hospitalization. Functional status at discharge was assessed with the Barthel Index (BI). Neuroimaging and vascular evaluations-including computed tomography, magnetic resonance imaging, echocardiography, electrocardiography, carotid ultrasonography, and transcranial Doppler-were performed to determine lesion location and classify stroke subtype.

Fasting venous blood samples were collected between 6:00 and 7:00 a.m. after an overnight fast of at least 8 h, within 24 h of hospital admission. The time from stroke onset to blood collection was recorded for each patient. Complete blood count analysis was conducted using an automated hematology analyzer (BZ6800, China) to quantify white blood cells (WBC), neutrophils (N), monocytes (M), platelets (P), and lymphocytes (L). Serum creatinine (Cr) and uric acid (UA) levels were also measured. Based on the cellular counts, the systemic immune-inflammation index (SII) and systemic inflammation response index (SIRI) were calculated as SII = P × (N/L) and SIRI = N × (M/L), respectively (29). All blood assays were performed in triplicate.

### Definition of early-onset PSD

Clinical assessments were performed by board-certified neurologists and psychiatrists who remained blinded to the study objectives. Early-onset post-stroke depression (PSD) was diagnosed according to the Diagnostic and Statistical Manual of Mental Disorders, Fifth Edition (DSM-5) criteria, with evaluations conducted at 2 weeks after acute ischemic stroke onset. The 17-item Hamilton Depression Rating Scale (HAMD-17) was used to quantify depressive symptom severity. According to the recommendation, HAMD-17 score of ≥7 was used as the cutoff to define early-onset PSD (30, 31). Patients with HAMD-17 scores <7 were assigned to the NPSD group, whereas those with scores ≥7 were classified into the early-onset PSD group. Based on established cutoffs, depression severity was further categorized as mild (HAMD-17 score 7–17), moderate (18–23), or severe (≥24) (32).

### Statistical analysis

All enrolled patients had complete data for the variables included in the analyses (demographics, vascular risk factors, laboratory parameters, NIHSS, Barthel Index, and HAMD-17). Patients with any missing data on these core variables were excluded prospectively based on our exclusion criteria. All statistical analyses were performed using SPSS version 25.0 (IBM Corp., Armonk, NY, United States). Data normality was assessed with the Kolmogorov–Smirnov test. Normally distributed continuous variables are expressed as mean ± standard deviation (SD), and non-normally distributed data as median with interquartile range (IQR). Categorical variables are presented as frequencies and percentages. Group comparisons were conducted using the Chi-square test or Fisher’s exact test for categorical variables, and the Student’s *t*-test or Mann–Whitney U test for continuous variables, as appropriate. The distributions of key variables across early-onset PSD severity categories were visualized using box plots. Spearman’s rank correlation analysis was employed to examine the relationships between inflammatory indices and HAMD-17 scores. Multicollinearity among independent variables was evaluated prior to regression modeling. Binary logistic regression was performed to identify risk factors associated with early-onset PSD. Sensitivity analyses were conducted to test the robustness of the primary findings. The discriminative ability of SII and SIRI for early-onset PSD was assessed using receiver operating characteristic (ROC) curve analysis. A two-tailed *p*-value < 0.05 was considered statistically significant.

## Results

### Clinical and demographic characteristics of NPSD and early-onset PSD groups

**Figure 2:**
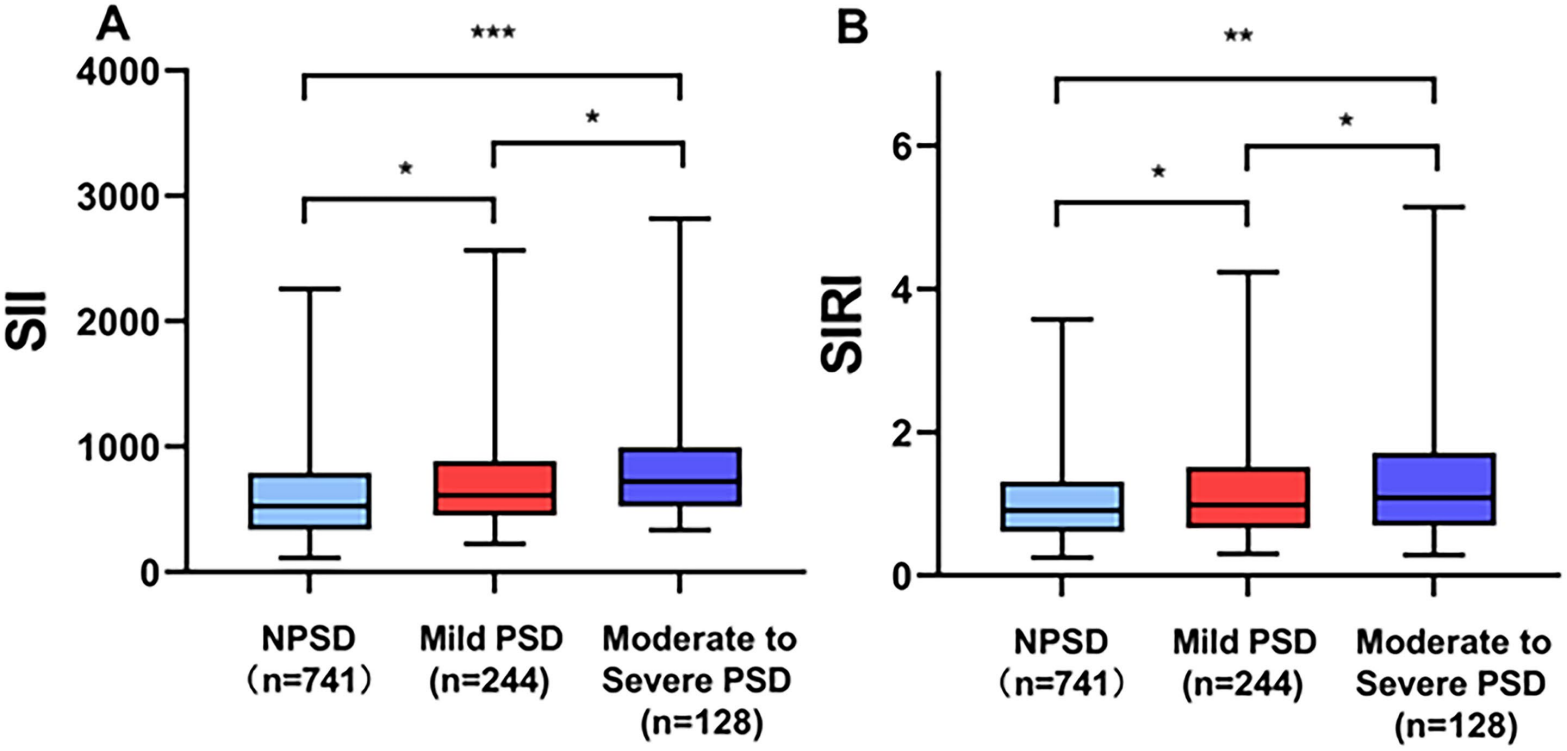
Comparison of **(A)** SII and **(B)** SIRI across different severity levels of early-onset PSD. ***p < 0.001, **p < 0.01, *p < 0.05.

Table 1 provides a comprehensive summary of the clinical and demographic characteristics of the study population. Of the 1,113 enrolled patients, 372 (33.42%) were diagnosed with early-onset post-stroke depression (PSD), while 741 (66.58%) comprised the non-PSD (NPSD) group. Comparative analyses revealed significant differences between the two groups. The early-onset PSD group had a significantly lower proportion of male patients (*p* < 0.001) and lower Barthel Index (BI) scores (*p* < 0.001) compared to the NPSD group. In contrast, the early-onset PSD group showed significantly higher values for age (*p* < 0.001), NIHSS score (*p* < 0.001), HAMD-17 score (*p* < 0.001), systemic immune-inflammation index (SII, *p* < 0.001), and systemic inflammation response index (SIRI, *p* = 0.004). Furthermore, the distributions of SII and SIRI across different severity levels of early-onset PSD are illustrated in Figure 2.

**Table 1:** Characteristics of patients in the NPSD and PSD groups.

Variable	Total (*n* = 1,113)	PSD (*n* = 372)	NPSD (*n* = 741)	*P*
Demographic characteristics				
Age, years	66.53 ± 11.94	67.52 ± 11.53	64.56 ± 12.50	<0.001
Male, *n* (%)	739 (66.40)	213 (57.26)	526 (70.99)	<0.001
BMI, kg/m^2^	24.10 ± 3.42	24.12 ± 3.43	23.98 ± 3.40	0.084
Treatment options				
Intravenous thrombolysis	125 (11.23)	39 (10.48)	86 (11.61)	0.576
Cerebral thrombectomy	52 (4.67)	17 (4.57)	35 (4.72)	0.094
Vascular risk factors, *n* (%)				
Current drinking	294 (26.42)	95 (25.54)	199 (26.86)	0.638
Current smoking	487 (43.76)	158 (42.47)	329 (44.40)	0.541
Diabetes mellitus	283 (25.43)	97 (26.08)	186 (25.10)	0.725
Hypertension	893 (80.23)	309 (83.06)	584 (78.81)	0.093
Coronary artery disease	179 (16.08)	69 (18.55)	110 (14.84)	0.113
History of medications, *n* (%)				
Previous antihypertension drugs	827 (74.30)	285 (76.61)	542 (73.14)	0.212
Previous hypoglycemic drugs	272 (24.44)	93 (25.00)	179 (24.16)	0.757
Previous antiplatelet drugs	170 (15.27)	50 (13.44)	120 (16.19)	0.228
Previous lipid-altering drugs	139 (12.49)	49 (13.17)	90 (12.16)	0.625
Stroke subtype, *n* (%)				0.136
LAA	294 (26.42)	99 (26.13)	195 (26.32)	
LI	514 (46.18)	174 (46.77)	340 (45.88)	
CE	202 (18.15)	68 (18.28)	134 (18.08)	
SOE	45 (4.04)	13 (3.49)	32 (4.32)	
SUE	59 (5.30)	18 (4.84)	41 (5.53)	
Neuropsychological evaluation				
NIHSS score, median (IQR)	4 (3–8)	5 (2–9)	3 (1–6)	<0.001
BI score, median (IQR)	86 (76–100)	81 (73–100)	95 (75–100)	<0.001
HAMD-17score, median (IQR)	6 (4–13)	17 (13–20)	5 (3–6)	<0.001
Lesion location, *n* (%)				
Frontal lobe	83 (7.46)	31 (8.33)	52 (7.02)	0.431
Temporal lobe	40 (3.59)	15 (4.03)	25 (3.37)	0.578
Parietal lobe	91 (8.18)	31 (8.33)	60 (8.10)	0.892
Occipital lobe	35 (3.14)	10 (2.69)	25 (3.37)	0.541
Basal ganglia	459 (41.24)	154 (41.40)	305 (41.16)	0.940
Thalamus	73 (6.56)	23 (6.18)	50 (6.75)	0.720
Brainstem	233 (20.93)	78 (20.97)	155 (20.92)	0.985
Cerebellum	100 (8.98)	30 (8.06)	70 (9.45)	0.447
Left hemisphere infarctions	527 (52.02)	178 (47.85)	349 (47.10)	0.112
Right hemisphere infarctions	486 (47.98)	165 (44.35)	321 (43.32)	0.743
Laboratory data				
Timing of blood collection, hours	36.5 (24.0–58.0)	37.0 (24.5–59.0)	36.0 (24.0–57.5)	0.482
WBC (×10^9^/L)	6.96 ± 1.88	6.98 ± 1.85	6.95 ± 1.89	0.758
Neutrophils (×10^9^/L)	4.4 (3.31–5.47)	4.37 (3.36–5.47)	4.40 (3.30–5.47)	0.663
Platelets (×10^9^/L)	205 (168–242)	209 (177.25–251)	202 (161–237)	0.632
Monocytes (×10^9^/L)	0.4 (0.31–0.5)	0.4 (0.32–0.51)	0.39 (0.3–0.48)	0.052
Lymphocytes (×10^9^/L)	1.65 (1.26–2.12)	1.61 (1.22–2.08)	1.69 (1.29–2.17)	0.092
UA (mmol/L)	331.31 ± 92.65	332.17 ± 92.86	330.88 ± 92.60	0.827
Cr(mmol/L)	74.84 ± 34.62	74.34 ± 36.84	75.10 ± 33.47	0.730
SII	524.47 (346–791)	535.48 (341–796)	520 (372.95–769)	<0.001
SIRI	1.03 (0.67–1.62)	1.07 (0.69–1.71)	0.94 (0.64–1.45)	0.004

NIHSS, National Institutes of Health Stroke Scale; BI, Barthel Index; HAMD-17, Hamilton depression scale 17 items; LAA, large-artery atherosclerosis; LI, lacunar infarction; CE, cardioembolism; SOE, stroke of other determined etiology; SUE, stroke of undetermined etiology; WBC, white blood cell; Cr, creatinine; UA, Uric acid; SII, systemic immune-inflammatory index; SIRI, systemic inflammation response index.

### Correlation between inflammatory indices and depression severity in all patients

**Figure 3:**
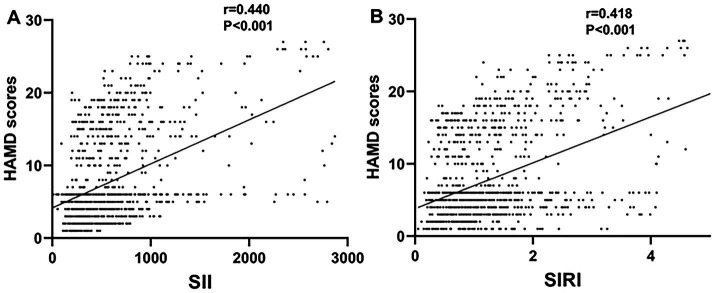
Scatter plots showing that the SII (*r* = 0.440, *p* < 0.001; **A**) and SIRI (*r* = 0.418, *p* < 0.001; **B**) were positively correlated with the HAMD scores.

As shown in Figure 3, HAMD-17 scores demonstrated significant positive correlations with both the SII (*r* = 0.440, *p* < 0.001) and the SIRI (*r* = 0.418, *p* < 0.001).

### Logistic regression analysis of risk factors for early-onset PSD

To identify independent risk factors for early-onset post-stroke depression (PSD), we performed binary logistic regression using variables that showed statistical significance in the univariate analyses (Table 1). Table 2 presents the results of models for early-onset PSD. Collinearity diagnostics using variance inflation factor (VIF) indicated no substantial multicollinearity between the SII (VIF = 1.51) and the SIRI (VIF = 1.43). After adjusting for all potential confounders, both the SII (OR, 1.762, 95% CI: 1.261–1.946, *p* < 0.001) and the SIRI (OR, 1.672, 95% CI: 1.348–1.932, *p* = 0.004) remained independent predictors of early-onset PSD. When SII and SIRI were analyzed as tertile-based categorical variables, patients in the highest tertile of SII (3rd quartile vs. 1st quartile; OR = 1.902, 95% CI: 1.513–2.281, *p* < 0.001) and those in the highest tertile of SIRI (3rd quartile vs. 1st quartile; OR = 1.855, 95% CI: 1.313–2.001, *p* = 0.005) continued to show a significantly increased risk of early-onset PSD after multivariable adjustment (Table 3).

**Table 2:** Logistic regression analysis for risk factors with early-onset PSD.

Variable	OR (95% CI)	*P*	Adjusted OR (95% CI)	*P*
Age	1.304 (1.005–1.642)	0.031	1.203 (1.021–1.484)	0.116
Male	0.701 (0.503–0.901)	0.002	0.913 (0.771–0.984)	0.093
NIHSS score	1.915 (1.666–2.113)	<0.001	1.804 (1.511–1.971)	0.076
BI score	1.553 (1.103–1.704)	0.006	1.331 (1.094–1.515)	0.213
SII	2.015 (1.313–2.275)	<0.001	1.762 (1.261–1.946)	<0.001
SIRI	1.885 (1.327–2.131)	<0.001	1.672 (1.348–1.932)	0.004

**Table 3:** Association between SII, SIRI and early-onset PSD.

Variable	OR (95% CI)	*P*	Adjusted OR (95% CI)^a^	*P*
SII ternary classification				
T1	Reference		Reference	
T2	1.692 (1.303–2.013)	<0.001	1.598 (1.221–1.801)	0.009
T3	2.226 (1.781–2.608)	<0.001	1.902 (1.513–2.281)	<0.001
SIRI ternary classification				
T1	Reference		Reference	
T2	1.608 (1.396–1.992)	<0.001	1.502 (1.209–1.788)	0.012
T3	2.061 (1.615–2.225)	<0.001	1.855 (1.313–2.001)	0.005

SII: T1 ≤ 425.84; 425.84 < T2 ≤ 663.90; T3 > 663.90. SIRI: T1 ≤ 0.85; 0.85 < T2 ≤ 1.40; T3 > 1.40.

a Model: adjusted for age, male, initial NIHSS score, and BI score.

### Subgroup analyses and interaction test

**Figure 4:**
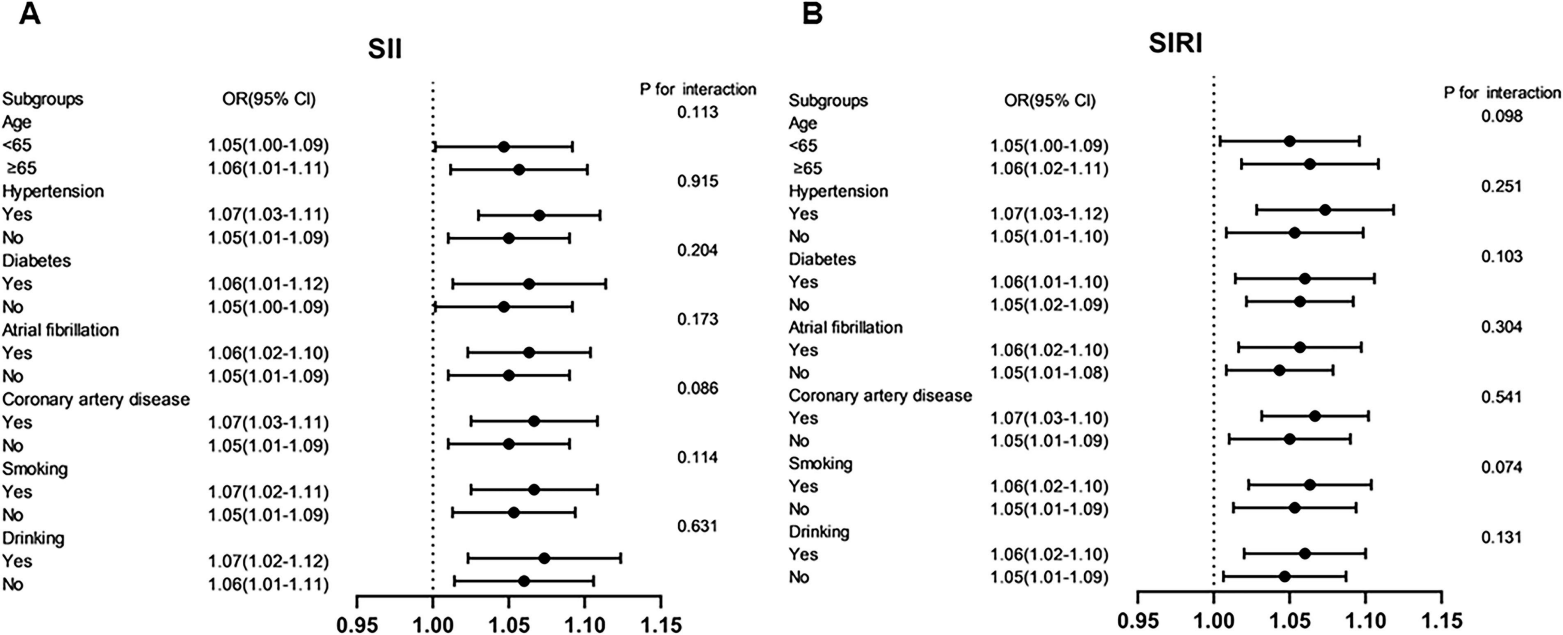
Subgroup analyses of the associations between the SII, SIRI, and early-onset PSD.

To assess the consistency of the associations between SII, SIRI, and early-onset PSD, we performed subgroup analyses across predefined demographic and clinical strata (Figure 4). The positive associations remained robust in all subgroups stratified by age (<65 vs. ≥65 years), hypertension, diabetes mellitus, atrial fibrillation, coronary artery disease, smoking status, and alcohol consumption. In comprehensive stratified analyses, both SII and SIRI maintained significant associations with early-onset PSD across all examined subgroups, supporting the robustness and generalizability of these relationships in diverse patient profiles. Formal interaction tests confirmed the absence of significant effect modification by any of the stratification variables (all P for interaction > 0.05).

### The diagnostic performance of the SII and SIRI for early-onset PSD was evaluated using ROC analysis

**Figure 5:**
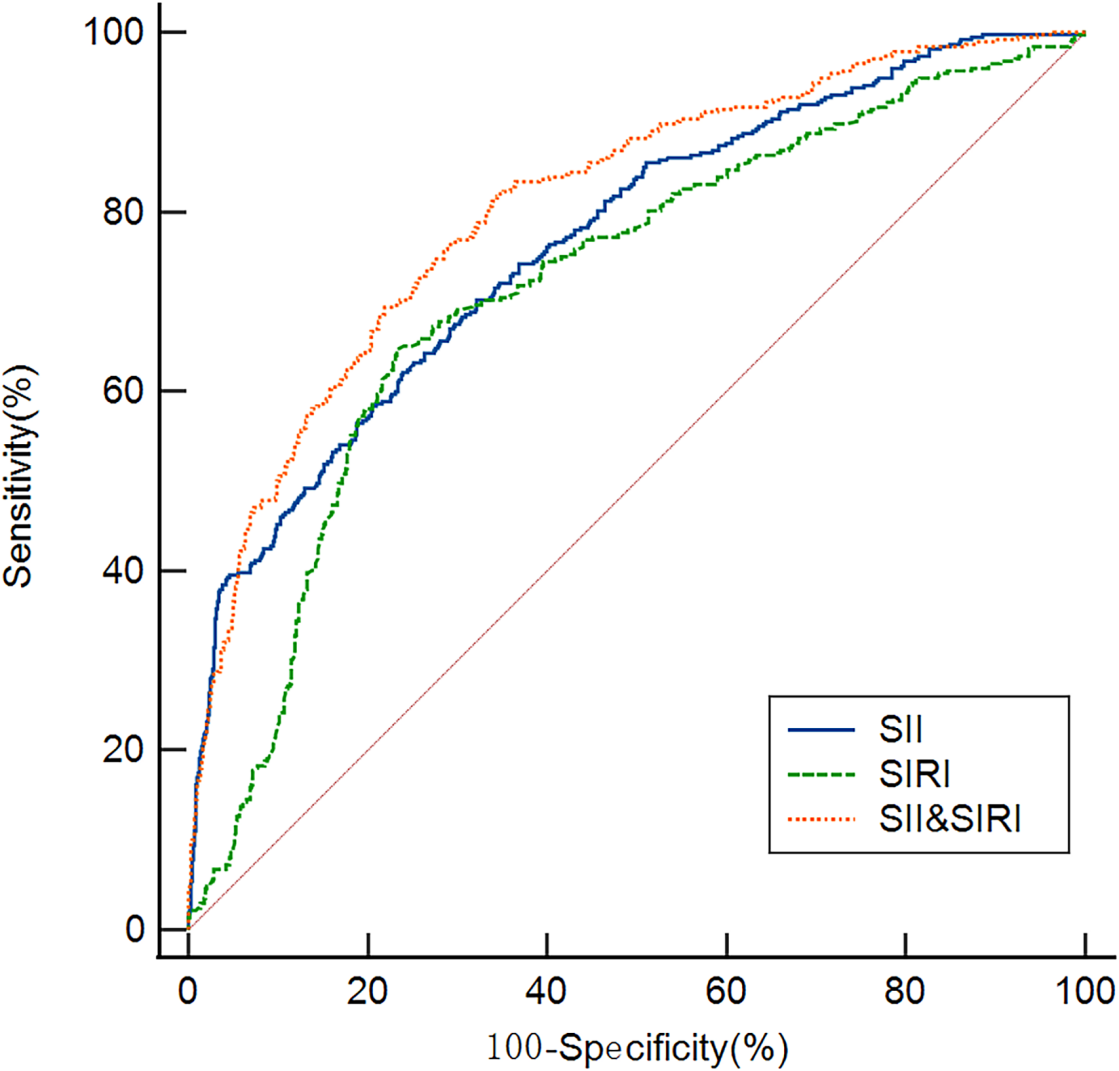
ROC curves demonstrating the discriminatory power of the SII, SIRI, and their combination for early-onset PSD. The AUC were 0.767 for the SII, 0.718 for the SIRI, and 0.807 for the combined model.

As shown in Figure 5, the SII achieved an AUC of 0.767 (95% CI: 0.741–0.791; *p* < 0.001), with an optimal cutoff value of 714 yielding a sensitivity of 62.10% and specificity of 76.11%. The SIRI demonstrated an AUC of 0.718 (95% CI: 0.691–0.744; *p* < 0.001), with a cutoff of 1.07 corresponding to 64.78% sensitivity and 76.52% specificity. Notably, the combination of SII and SIRI significantly enhanced predictive accuracy, achieving an AUC of 0.807 (95% CI: 0.783–0.830; *p* < 0.001). At the optimal combined cutoff of 0.31, sensitivity reached 69.35% with specificity of 78.14%. These results demonstrate that although both indices alone exhibit significant discriminative ability for early-onset PSD, their combined use offers superior diagnostic performance.

### Incremental predictive value of SII and SIRI beyond clinical factors

To evaluate whether SII and SIRI provide additional prognostic information beyond established clinical predictors (age, sex, NIHSS, and BI), we performed ROC and reclassification analyses. The clinical model alone achieved an AUC of 0.712 (95% CI: 0.684–0.740). Adding SII significantly increased the AUC to 0.785 (95% CI: 0.759–0.811, *p* < 0.001), with an net reclassification improvement (NRI) of 0.182 (95% CI: 0.094–0.270) and integrated discrimination improvement (IDI) of 0.053 (95% CI: 0.031–0.075). Adding SIRI also improved the AUC to 0.749 (95% CI: 0.722–0.776, *p* = 0.008). The combination of SII and SIRI yielded the highest AUC of 0.807 (95% CI: 0.783–0.830, *p* < 0.001), with an NRI of 0.231 and IDI of 0.072. These results indicate that SII and SIRI offer modest but statistically significant incremental predictive value for early-onset PSD beyond traditional clinical variables.

## Discussion

The pathophysiology of PSD involves multifactorial interactions, including neurotransmitter disturbances (insufficient secretion of serotonin and norepinephrine), neuroinflammation (pro-inflammatory cytokines impairing limbic system function), and reduced brain-derived neurotrophic factor leading to diminished synaptic plasticity (7). Given the growing evidence implicating inflammatory mechanisms in early-onset PSD, we investigated the SII and SIRI as potential biomarkers. Our study yielded several key findings. Compared with the NPSD group, patients with early-onset PSD exhibited significantly higher SII, SIRI, and NIHSS scores, along with lower BI scores. HAMD-17 scores were positively correlated with both SII and SIRI levels. Logistic regression identified SII and SIRI as independent risk factors for early-onset PSD. Subgroup analyses revealed no significant interactions, indicating that the associations of SII and SIRI with early-onset PSD were consistent across different demographic and clinical strata. Furthermore, ROC analysis demonstrated clinically meaningful discriminative ability of SII and SIRI for early-onset PSD. Collectively, these results establish a significant correlation between elevated SII and SIRI levels and early-onset PSD, supporting their potential utility as inflammatory biomarkers in this clinical context.

The SII, which integrates neutrophil, lymphocyte, and platelet counts, serves as a novel composite biomarker reflecting systemic inflammatory and immune status (33). In our cohort, 372 patients were diagnosed with early-onset PSD, a prevalence consistent with previous reports (1, 3, 4). Prior evidence indicates that stroke survivors who develop depression within 3 months post-stroke are at high risk of persistent depressive symptoms and account for approximately two-thirds of incident cases during the first year after stroke (34), underscoring the need for ongoing clinical monitoring in this population. Compared with the NPSD group, patients with early-onset PSD had more severe stroke severity and functional disability. Such unfavorable physical conditions may exacerbate depressive symptoms and other psychological complications (35). Consistent with our findings, female stroke patients have been reported to be more susceptible to PSD than males (36, 37), and tend to exhibit more severe “reactive” depressive symptoms following stroke (38). SII showed a positive correlation with early-onset PSD severity. Binary logistic regression identified SII as an independent predictor of early-onset PSD. When analyzed as a tertile-based categorical variable, elevated SII remained an independent risk factor, aligning with earlier studies (21, 22). Subgroup analyses revealed no significant interaction effects, indicating that the association between SII and early-onset PSD was consistent across diverse demographic and clinical strata, thereby supporting the robustness and generalizability of our conclusions. Although previous research has linked gender, age, lesion location, and stroke severity to post-stroke depression (39), our study did not find significant associations between gender, age, or lesion location and early-onset PSD. We propose that discrepancies across studies may stem from differences in ethnic composition, sample size, medication profiles, and disease-severity distributions. Finally, SII demonstrated clinically meaningful discriminative performance for early-onset PSD. These data suggest that SII may serve as a useful biomarker for identifying early-onset PSD. Our findings enhance the understanding of SII’s role in early-onset PSD and provide new insights for future therapeutic strategies.

The SIRI is an emerging composite biomarker reflecting systemic inflammation and immune activity. Accumulating evidence links SIRI to prognosis in pneumonia, rheumatoid arthritis, and acute pancreatitis (12, 40, 41), as well as to cardiovascular risk stratification, where it demonstrates high predictive value for incident cardiovascular diseases (42–45). In coronary heart disease patients, SIRI correlates positively with the degree of coronary stenosis, serving as a potential early screening tool for assessing stenosis severity (46). Moreover, SIRI has been associated with all-cause mortality in stroke populations and outperforms traditional inflammatory markers-such as neutrophil-to-lymphocyte ratio, platelet-to-lymphocyte ratio, lymphocyte-to-monocyte ratio, and red cell distribution width-in predictive accuracy (25). A meta-analysis further supports the correlation between SIRI and adverse outcomes in stroke patients (47). Despite its growing recognition across clinical disciplines, the relationship between SIRI and early-onset PSD remains poorly characterized. To our knowledge, this is the first study to systematically examine the predictive utility of SIRI for early-onset PSD. Our findings revealed significantly elevated SIRI levels in patients with early-onset PSD compared to NPSD controls. HAMD-17 scores showed a positive correlation with SIRI levels, and logistic regression confirmed SIRI as an independent risk factor for early-onset PSD. Additionally, SIRI exhibited clinically relevant discriminative capacity. These results expand current understanding of SIRI’s role in early-onset PSD and may inform the development of novel therapeutic approaches.

The interaction between inflammation and PSD has been increasingly substantiated by accumulating research. Both the SII and SIRI are novel composite markers of inflammation. Our study demonstrates that SII and SIRI serve as independent risk factors for early-onset PSD and may represent potential tools for its identification. The underlying mechanism by which SII and SIRI increases the risk of early-onset PSD remains incompletely understood. However, several potential pathophysiological pathways may help explain this association. Previous research indicated that responses to inflammation in many central and peripheral systems may be biological factors contributing to PSD (48, 49). During AIS, BBB disruption is accompanied by enhanced brain edema, in which neutrophils are initially produced in the infarct core and penumbra regions (50). They release inflammatory factors that damage endothelial cells and basement membranes, respectively (51). Monocytes migrate early into the injured area and differentiate into macrophages in response to oxidized low-density lipoprotein, capable of phagocytosing low density lipoprotein and forming foam cells that release various inflammatory factors (52). Additionally, activated monocytes secrete vascular endothelial growth factor, which promotes vascular permeability and further compromises BBB integrity (53). Concurrently, lymphocyte concentrations rise following ischemic stroke and play a pivotal role in AIS pathogenesis by modulating neuroinflammation through the production of pro-inflammatory cytokines and post-stroke cytotoxic effects (54). Lymphocyte-endothelial interactions can exacerbate microvascular dysfunction and inflammatory mediator release, leading to neuronal death and BBB disruption (55). Among T-lymphocyte subsets, helper T cells (CD4^+^ T cells) have emerged as biomarkers for predicting stroke outcomes; they act as immunomodulators after stroke and may exert protective effects against inflammatory brain injury (56). Cytotoxic CD8^+^ T cells also mount a robust response post-stroke, expanding rapidly and persisting at elevated levels, with stroke-related neuroinflammation potentially altering their subset distribution (57). Increases in certain T-cell subsets have been linked to recurrence and mortality after ischemic stroke (58). Furthermore, activated platelets exhibit enhanced rolling and adhesion to vascular endothelial cells, altering endothelial function and promoting neutrophil chemotaxis (59). Collectively, these inflammatory markers disrupt neurotransmission and contribute to lasting cerebral dysfunction, including depression and cognitive impairment (60, 61).

Given the substantial clinical and pathological heterogeneity observed in early-onset PSD, reliable risk prediction and progression monitoring are unlikely to be achieved through any single biomarker. Consequently, the integration of complementary biomarkers is essential for enhancing the accuracy of early-onset PSD risk estimation. Our ROC analyses showed that SII and SIRI alone have moderate discriminatory ability for early-onset PSD (AUCs of 0.767 and 0.718, respectively), which is not sufficient to recommend them as standalone screening or diagnostic tools. However, when added to a clinical model consisting of age, sex, NIHSS, and functional status, both indices significantly improved the AUC, NRI, and IDI, with the combined SII + SIRI model achieving an AUC of 0.807. These findings suggest that SII and SIRI may serve as useful adjunctive biomarkers to enhance risk stratification, particularly in patients with minor stroke. Nevertheless, the absolute improvement in discrimination was modest, and external validation in larger, multicenter cohorts is needed before clinical implementation.

This study has several limitations that should be considered when interpreting the findings: (1) Several important psychosocial factors associated with post-stroke depression, including educational level, socioeconomic status, marital status, social support, sleep quality, and previous psychiatric symptoms, were not assessed. These variables may independently affect both systemic inflammation and depressive symptoms, potentially introducing residual confounding. Therefore, our findings should be interpreted as hypothesis-generating, and future studies should incorporate comprehensive psychosocial assessments. (2) The exclusion of patients with severe aphasia, coma, or dementia during hospitalization may have introduced selection bias and limited the representation of severe stroke cases. (3) Because SII and SIRI are composite indices derived from neutrophil, monocyte, platelet, and lymphocyte counts-each of which may independently contribute to early-onset PSD-their interpretation requires caution. Larger, multi-center studies are warranted to confirm their independent clinical utility. (4) Future studies should integrate a broader panel of circulating inflammatory biomarkers to refine risk stratification and elucidate underlying causal pathways. (5) The observational nature and relatively short follow-up period limit the ability to draw longitudinal or causal inferences; longer-term prospective studies are recommended. (6) Thyroid function tests were not routinely measured in our cohort. Thyroid dysfunction is known to influence both systemic inflammation and mood disorders, and its absence from our analysis may introduce residual confounding. Future studies should incorporate thyroid function assessment to more rigorously evaluate the independent associations of SII and SIRI with early-onset PSD. (7) We did not systematically record the presence of infections (e.g., pneumonia, urinary tract infection) at admission or during follow-up. Infections can elevate SII and SIRI levels and potentially influence depression risk. Although WBC counts did not differ between groups and blood was collected early after stroke onset before most infections typically manifest, residual confounding by subclinical infection cannot be excluded. Future studies should include comprehensive infection assessments to validate our findings. (8) As a single-center study conducted exclusively in a Chinese population, the results may be subject to inherent demographic and regional biases; multicenter validation across diverse geographic and ethnic cohorts is needed to enhance external validity. More importantly, our study exclusively enrolled patients with minor acute ischemic stroke. Patients with severe strokes, impaired consciousness, aphasia, or dementia were excluded due to feasibility constraints. Consequently, our findings and conclusions are primarily applicable to patients with minor acute ischemic stroke. Future studies should investigate the role of SII and SIRI in early-onset PSD across the full spectrum of stroke severity.

## Conclusion

Our findings suggest that the SII and SIRI may serve as predictive biomarkers and independent risk factors for early-onset PSD. Establishing a clear association between these inflammatory indices and early-onset PSD would carry significant clinical implications, including improved screening accuracy, more tailored treatment planning, enhanced etiological understanding, and refined prognostic evaluation. Integrating SII and SIRI into routine clinical assessment and therapeutic strategies could contribute to better overall outcomes for affected patients. Nevertheless, further research is essential to precisely delineate the pathophysiological roles of SII and SIRI in early-onset PSD.

## Data Availability

The raw data supporting the conclusions of this article will be made available by the authors, without undue reservation.
